# Ovarian Cancer and the Microbiome: Connecting the Dots for Early Diagnosis and Therapeutic Innovations—A Review

**DOI:** 10.3390/medicina60030516

**Published:** 2024-03-21

**Authors:** Seo-Yoon Choi, Jung-Hye Choi

**Affiliations:** College of Pharmacy, Kyung Hee University, Seoul 02447, Republic of Korea; okc7399@khu.ac.kr

**Keywords:** ovarian cancer, microbiome, inflammation, endometriosis, pelvic inflammatory disease, chlamydia

## Abstract

Ovarian cancer, which ranks eighth among global female cancers and fifth in fatality, poses a significant health challenge owing to its asymptomatic early stages. Understanding the pathogenesis requires extensive research. Recent studies have emphasized the role of the gut and cervicovaginal microbiota in ovarian cancer. This review explores the current understanding of the relationship between the microbiome and ovarian cancer, considering the potential of biomarkers in the serum and various tissues. Insights into the influence of the microbiome on treatments, including surgery and chemotherapy, open doors to innovative approaches, such as fecal microbiome transplantation. This synthesis of recent findings provides crucial insights into the intricate interplay between the microbiome and ovarian cancer, thereby shaping diagnostic and treatment strategies.

## 1. Introduction

The term “microbiome” encompasses the complete genetic information of the microorganisms inhabiting the human body, surpassing eight million species, which is 360 times more extensive than the human genome [1]. Under normal conditions, indigenous microbiota thrive in diverse regions of the human body, with at least 10,000 species [1]. Notably, the skin hosts approximately 500 species of bacteria and yeast, whereas the gastrointestinal microbiome comprises 500–1000 species, including bacteria, fungi, archaea, and protozoa [1]. Indigenous microbiota reside in areas such as the upper respiratory tract, external genitalia, and vagina [1]. The microbiome plays a pivotal role in various physiological and pathological processes and is intricately linked to the immune system [2]. Recent studies have revealed associations between sleep disorders [3], depression [3], aging [4], and cancer.

Certain microbes, such as *Helicobacter pylori* in the stomach and *Fusobacterium nucleatum* in the colon, have been identified as contributors to the risk and progression of stomach cancer and colorectal cancers, respectively [5]. Moreover, studies have revealed that microbiome dysbiosis, even in distant regions, influences cancer progression; for instance, gut microbiota dysbiosis affects hepatocellular carcinoma progression [6] and contributes to breast cancer development [7].

According to the 2020 GLOBOCAN Global Cancer Women’s Cancer Data, ovarian cancer has an incidence rate of 3.4% and mortality rate of 4.7% [8]. Each year, over 300,000 women develop ovarian cancer, which causes approximately 152,000 fatalities. Ovarian cancer is the most lethal gynecological cancer, ranking eighth in global female cancer incidence and fifth in terms of mortality. This high mortality rate is attributed to frequent late-stage diagnoses due to the absence of specific symptoms or definitive diagnostic biomarkers. Although risk factors, such as family history, hyperovulation, endometriosis, and dietary habits, have been known, much remains unclear [9]. Despite its low survival rate, a comprehensive understanding of ovarian cancer remains elusive and warrants further research.

Against this backdrop, studies exploring the nexus between the microbiome and ovarian cancer have recently emerged, aligning with analogous investigations on other cancer types. The cervical vaginal microbiome, which is close to the ovaries, and the gut microbiome, which is already known to be associated with various cancers, are of particular interest. This article reviews the latest discoveries regarding the connection between the microbiome and ovarian cancer, and delves into its potential applications in ovarian cancer diagnosis and treatment.

## 2. Microbiome and Ovarian Cancer

### 2.1. Gut Microbiome and Ovarian Cancer

#### 2.1.1. Inflammation

**Figure 1:**
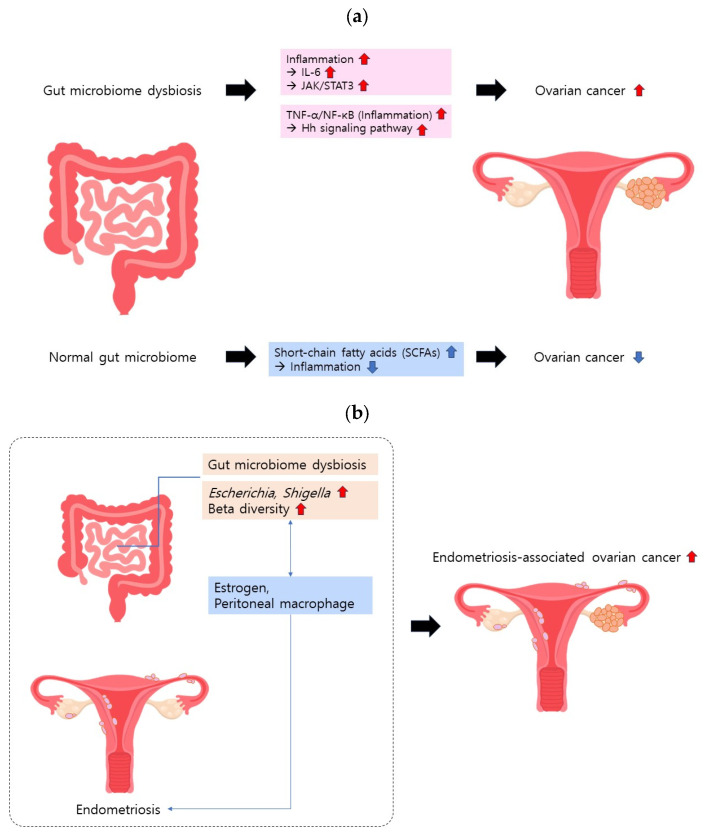
Gut microbiome and ovarian cancer. (**a**) Gut microbiome dysbiosis intensifies the inflammatory response, elevating the risk of ovarian cancer. (**b**) Gut microbiome dysbiosis exacerbates endometriosis, contributing to the development of ovarian cancer. Estrogen and peritoneal macrophages may be involved in endometriosis-associated ovarian cancer development.

Continued research has underscored the intricate relationship between the gut microbiome and inflammation [10,11,12,13]. One study proposed that the gut microbiome dampens inflammatory responses by influencing immune regulatory cells [11]. Conversely, another study indicated that the gut microbiome may modulate intestinal permeability, thereby intensifying inflammatory responses [12]. Increased intestinal permeability allows microbial products to enter the bloodstream, thereby triggering elevated levels of cytokines and other inflammatory mediators. Other studies have highlighted the anti-inflammatory and immunomodulatory properties of short-chain fatty acids (SCFAs), which are metabolites produced by specific gut microbes [14]. Additionally, studies have suggested that gut microbiota-derived bile acids are involved in inflammation [15]. Remarkably, inflammatory cytokines have been implicated in ovarian cancer [16,17,18] (Figure 1a). A cytokine of particular interest in the gut microbiome is interleukin-6 (IL-6), found to be elevated in the ovarian cancer microenvironment [16]. IL-6 activates the Janus tyrosine kinase/signal transducer and activator of the transcription 3 (JAK/STAT3) pathway, and STAT3 modifies the transcription of several genes promoting cancer [16]. Therefore, IL-6 may promote the development of high-grade ovarian cancer. Notably, Toll-like receptor 5 (TLR5) and the microbiome play crucial roles in IL-6 activation. Rutkowski et al. induced cancer in TLR5-deficient and TLR5-responsive mice via *p53* and K-ras gene mutations [19]. Despite similar cancer sizes, TLR5-responsive mice exhibited significantly higher serum IL-6 levels, and ovarian cancer progressed more rapidly than in the TLR5-dificient mice. Differences in microbiome composition, particularly in the *Allobaculum*, *Bacteroides*, and *Lactobacillus* genera, persisted even when the two groups shared the same cage, suggesting that TLR5 influences microbiome composition. Antibiotic treatment eliminating symbiotic bacteria erased the difference in serum IL-6 levels and dissimilarity in tumor growth, affirming the impact of the microbiome on ovarian cancer. Some studies have proposed that inflammatory responses linked to the gut microbiome may influence ovarian cancer carcinogenesis and progression through Hedgehog (Hh) signaling. The Hh signaling pathway has been recognized for its role in epithelial ovarian cancer (EOC) carcinogenesis and progression [20]. Tumor necrosis factor-α (TNF-α), a pro-inflammatory cytokine, has been identified as an activator of Hh signaling through nuclear factor kappa B (NF-κB) activation [21]. Hu et al. demonstrated that the gut microbiome from patients with EOC significantly increased serum TNF-α expression, up-regulated NF-κB, and activated Hh signaling, markedly enhancing EOC development [22]. These findings suggest that the gut microbiome from patients with EOC activates NF-kB, subsequently activating the Hh signaling pathway, potentially contributing to EOC development.

#### 2.1.2. Endometriosis

Numerous studies have reported an elevated risk of specific ovarian cancers in women with endometriosis compared to the general population [23,24]. These cancers are classified as endometriosis-associated ovarian carcinomas (EAOCs) and include clear-cell carcinomas, endometrioid ovarian carcinomas, and seromucinous borderline tumors [25]. A clear-cell carcinoma is associated with *PIK3CA* and *ARID1A* mutations, whereas endometrioid ovarian cancer is associated with mutations in *CTNNB1*, *PTEN*, and *ARID1A* mutations [24]. Mutations in *PTEN* [26,27] and *ARID1A* [21,28] have been identified in endometriosis. Furthermore, hepatocyte nuclear factor-1β (HNF-1β) upregulation has been observed in both ovarian clear-cell carcinoma and endometriosis [24,29,30], implying a connection between endometriosis and specific ovarian cancers. However, the exact association between endometriosis and EAOC remains unclear.

Endometriosis may be linked to dysbiosis of the gut microbiome (Figure 1b). In contrast to women without endometriosis, the gut microbiome of patients with endometriosis was dominated by *Escherichia* and *Shigella* [31]. A systematic review revealed that individuals with endometriosis had a higher abundance of *Actinobacteria*, *Firmicutes*, *Proteobacteria*, and *Verrucomicrobia* in their gut microbiomes than healthy individuals [32]. Conversely, the abundance of *Lactobacilli* was lower in individuals with endometriosis. A study in rats demonstrated that 42 days after inducing endometriosis, while the alpha diversity of the gut microbiome remained similar, the beta diversity increased [33]. The treatment of these rats with antibiotics reduced endometriotic lesions, and the oral administration of feces reversed this condition.

The precise mechanism linking endometriosis and the gut microbiome remains elusive and presents a complex and multifaceted interplay. Estrogen, which is recognized for its association with the development of endometriosis, is a key player in this interaction [34]. Several studies have proposed a bidirectional relationship between the gut microbiome and estrogen levels in estrogen-related diseases, suggesting the plausible involvement of the gut microbiome in the development or manifestation of endometriosis symptoms [35]. Peritoneal macrophages represent another avenue of exploration for understanding the intricate relationship between endometriosis and the gut microbiome. A previous study revealed that inflammation exacerbated by an abnormal gut microbiome has discernible effects on peritoneal macrophages [36]. Specifically, mice with an altered microbiome exhibit heightened intestinal permeability and leakage of bacterial products, contributing to macrophage dysregulation and an increased likelihood of persistent endometriosis. Conversely, Miller et al. proposed an alternative perspective, suggesting that peritoneal macrophages may exacerbate inflammation [37]. In their study, mice with endometriosis were treated with IL-17, a cytokine known to be elevated during endometriosis, resulting in an increase in the number of M2 peritoneal macrophages. Miller et al. further noted that M2 macrophages can potentially worsen endometriosis.

Collectively, these studies underscore the intricate interplay among inflammation, peritoneal macrophages, and the gut microbiome, which potentially elevates the risk of ovarian cancer by exacerbating endometriosis. However, the precise mechanisms governing this interaction remain unclear. Further research is imperative to unravel the exact mechanisms underlying the interaction between endometriosis and gut microbiome dysbiosis to reveal potential therapeutic targets and interventions.

### 2.2. Cervicovaginal Microbiome and Ovarian Cancer

#### 2.2.1. Chlamydia

**Figure 2:**
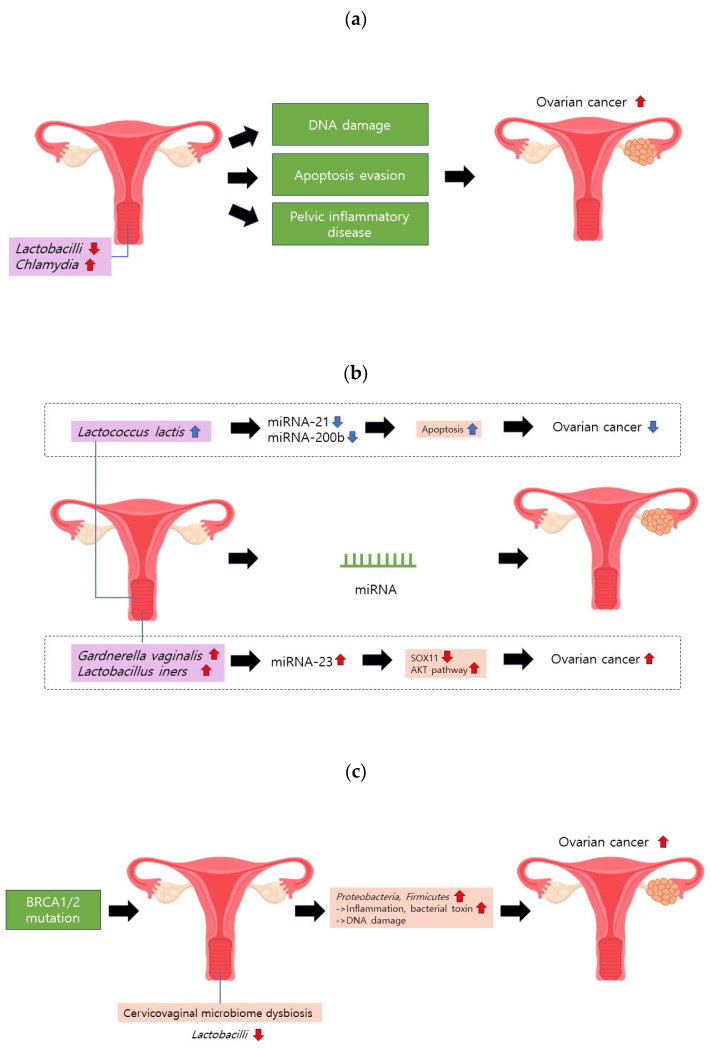
Cervicovaginal microbiome and ovarian cancer. (**a**) A reduction in *Lactobacilli* or an increase in *Chlamydia* may induce DNA damage, hinder apoptosis, and contribute to pelvic inflammatory disease (PID). These factors collectively elevate the risk of ovarian cancer. (**b**) *Gardnerella vaginalis* and *Lactobacillus iners* can upregulate miRNA-223. This molecular alteration may heighten ovarian cancer risk by diminishing *SOX11* expression and activating the AKT pathway. (**c**) BRCA1/2 mutations can instigate cervicovaginal microbiome dysbiosis, characterized by a decrease in *Lactobacilli* proportion.

Chlamydia resulting from a *Chlamydia trachomatis* infection, a prevalent sexually transmitted disease, has been linked to an increased risk of ovarian cancer (Figure 2a). Various studies have identified a high incidence of *Chlamydia* in ovarian cancer cells [38,39]. Seropositivity for the chlamydial plasmid-encoded Pgp3 antibody is associated with a two-fold higher risk of ovarian cancer [40]. Recent meta-analyses have further substantiated the correlation between chlamydial infections and ovarian cancer risk [41].

Two primary mechanisms have been proposed to explain how Chlamydia increases the risk of ovarian cancer. First, it induces DNA damage through the production of reactive oxygen species, concurrently impeding the base-excision repair pathway [42]. Second, Chlamydia evades apoptosis by inhibiting the release of mitochondrial caspase 3 and cytochrome C while downregulating p53 [39,42].

Studies have indicated that *Chlamydia* can ascend to the upper female genital tract, inducing inflammation and damage, potentially leading to pelvic inflammatory disease (PID) and an increased risk of ovarian cancer [43,44,45,46]. Certain *Lactobacillus* strains, including *Lactobacillus crispatus*, have demonstrated significant bactericidal effects against *Chlamydia trachomatis* [47]. In particular, *Lactobacillus crispatus* exhibits 90% bactericidal activity, mainly attributed to lactic acid production [47,48,49]. This suggests a potential risk reduction for ovarian cancer through the modulation of *Chlamydia* by *Lactobacillus*.

#### 2.2.2. Pelvic Inflammatory Disease

Chronic infections arising from PID contribute to the release of tumor-promoting substances such as cytokines, chemokines, and reactive oxygen species, fostering genetic and epigenetic alterations associated with cancer development [46]. However, there is an ongoing debate regarding the correlation between PID and ovarian cancer risk, with some studies reporting no significant association [50,51,52].

Ethnic disparities may contribute to conflicting findings. Zhou et al. noted that PID increased ovarian cancer risk in Asian women, but not in Caucasian women, possibly due to variations in lifestyle and oral contraceptive use [53]. Further complicating matters include inconsistencies within the same ethnic group, as demonstrated by the varying results of Taiwanese studies [52,54]. More comprehensive research controlling for numerous factors is warranted to ascertain the true relationship between PID and ovarian cancer risk.

#### 2.2.3. MicroRNAs

MicroRNAs (miRNAs) are short endogenous noncoding RNAs (18–25 nt) that have recently gained attention for their association with various cancers, including ovarian cancer [55,56,57,58]. The cervicovaginal microbiome may influence miRNA expression, potentially contributing to the OC development of ovarian cancer (Figure 2b).

In a study by Anton et al., *Gardnerella vaginalis*, which is implicated in bacterial vaginosis, upregulated miRNA-15a, miRNA-143, miRNA-145, miRNA-146, miRNA-223, and miRNA-148 [59]. Furthermore, *Lactobacillus iners*, which is linked to bacterial vaginosis and adverse pregnancy outcomes, upregulates miRNA-146, miRNA-193b, and miRNA-223 [59,60]. Notably, miRNA-223 is overexpressed in ovarian cancer cells and the serum exosomes of patients with EOC [61,62]. miRNA-223-3p overexpression is associated with reduced levels of the sex-determining region Y-box 11 (SOX11) [61]. They also found that SOX11 overexpression inhibits the growth, migration, and invasion of ovarian cancer cells. Another study has suggested that miRNA-223 overexpression promotes ovarian cancer development by activating the AKT pathway [62]. Conversely, Pan et al. reported the downregulation of miRNA-223 in the exosomes of patients with EOC [63]. Given these conflicting findings, further investigations are needed to clarify the role of miRNA-223 in EOC pathogenesis. 

Saadat et al. demonstrated that *Lactococcus lactis*, a probiotic from the vagina, reduced the expression of miRNA-21 and miRNA-200b and enhanced apoptosis in ovarian cancer cells [64]. The suppression of miRNA-21 has been linked to reduced cancer cell proliferation and tumor growth, whereas the miRNA-200 family plays a role in the initiation and progression of ovarian cancer.

Although promising, the understanding of miRNA–microbiome interactions in ovarian cancer remains limited, necessitating further research to unravel specific miRNA alterations and their implications.

#### 2.2.4. BRCA Mutation

Mutations in *BRCA1/2* substantially increase the risk of ovarian cancer, with a risk range of 39–63% in BRCA1 mutation carriers and 16.5–27% in *BRCA2* mutation carriers [65,66,67]. Intriguingly, one study proposed the potential interplay between BRCA1/2 mutations and the cervicovaginal microbiome, which influences the risk of ovarian cancer [68] (Figure 2c). In particular, women under 50 years of age with *BRCA1* mutations exhibit a reduced proportion of *Lactobacilli* in their cervicovaginal microbiomes compared to those without mutations. This reduction in *Lactobacilli* was consistent across disease stages, suggesting a potential causal relationship. Nené et al. introduced mechanistic insights into this decrease in *Lactobacilli*, linking it to an increase in the progesterone concentration during the luteal phase, which reduced the vaginal glycogen concentration [68]. Given that *Lactobacilli* thrive in the glycogen metabolism, this unfavorable condition may have contributed to their decrease [68]. Moreover, the evidence suggests that a reduction in *Lactobacilli* may play a causal role in the development of ovarian cancer. *Proteobacteria* and *Firmicutes*, which were predominant in cancer samples, can induce inflammation, release bacterial toxins that damage DNA, and contribute to the development of cancer.

In contrast, progesterone, known for its inhibitory effects on ovarian cancer [69,70,71], raises questions regarding the significance of its role in reducing *Lactobacilli* and impacting ovarian cancer risk in women with *BRCA* mutations. Increased progesterone levels in these women may contribute to a decrease in *Lactobacilli*; however, their impact on ovarian cancer risk remains uncertain. As Nené et al. acknowledged, it is plausible that factors beyond increased progesterone levels may influence the reduction in *Lactobacilli* and the subsequent increase in ovarian cancer risk in women with *BRCA* mutations, necessitating further research for a comprehensive understanding of these intricate interactions.

## 3. Microbiome and the Diagnosis and Treatment of Ovarian Cancer

### 3.1. Ovarian Cancer Diagnosis and Microbiome Markers

Ovarian cancer has a commendable five-year survival rate, exceeding 90% when diagnosed at stage I [72]. The urgency for an early diagnosis is underscored by the advanced stage at which ovarian cancer is detected. However, the widely employed ovarian cancer biomarker cancer antigen 125 lacks specificity owing to its elevated levels in conditions such as endometriosis and other cancers [73,74]. Hence, it is imperative to establish novel and specific biomarkers for ovarian cancer diagnosis.

Given its potential implications, the microbiome has emerged as a candidate contributor to ovarian cancer development. Although the causative or consequential nature of this association remains unclear, studies have consistently reported altered microbiome compositions in various body sites in patients with ovarian cancer compared to healthy individuals, suggesting a potential role of the microbiome as a diagnostic biomarker (Table 1). One study demonstrated elevated *Bacteroides*, *Prevotella*, and *Proteobacteria* as well as reduced levels of *Ruminococcus* and *Actinobacteria* in patients with ovarian cancer [75]. Of note, *Prevotella*, linked to a proinflammatory state, is associated with cervical and endometrial cancers [76,77]. However, the prospects of using the gut microbiome as a biomarker require further investigation. Miao et al. investigated the peritoneal microbiome and revealed reduced microbial diversity and a distinctive microbial signature in patients with ovarian cancer compared to those with benign adnexal masses [78]. Of these, 18 clusters were highly specific to ovarian cancer pathology.

Another study identified the presence of *Brucella*, *Chlamydia*, and *Mycoplasma* in ovarian cancer tumor cells, with *Brucella* believed to drive pelvic inflammation, leading to ovarian cancer [38,79]. *Chlamydia* significantly increases the risk of ovarian cancer, although further research is needed to elucidate *Mycoplasma*’s role [41]. Additionally, investigations into the dominant taxa in ovarian cancer cells revealed an increased *Proteobacteria*/*Firmicutes* ratio and significant changes in specific bacteria such as *Acinetobacter* and *Lactococcus* [80]. Notably, the decrease in *Lactococcus*, a probiotic candidate, and alterations in antibacterial response genes suggest potential microbiota-driven biomarkers for ovarian cancer.

Studies have consistently reported decreased levels of *Lactobacilli* in the cervicovaginal microbiome of patients with ovarian cancer [68,72,75]. *Lactobacilli* alone may have limited diagnostic value because they are also reduced in certain cervical cancer types; however, when combined with other biomarkers, they can increase their diagnostic accuracy [81]. Noteworthy is a study by Asangba et al., revealing distinct patterns of bacteria associated with different stages of ovarian cancer, offering potential insights into early diagnosis and prognosis [82].

In serum microbiome studies, *Acinetobacter*’s increased abundance in patients with ovarian cancer, as seen in the studies by Zhou et al. [80] and Kim et al. [83], aligns with its potential diagnostic significance. Serum samples, which are less invasive and more easily obtainable, represent a promising avenue for microbiome-based biomarker studies.

Exploiting the microbiome as a diagnostic tool holds promise, given its noninvasive nature and compatibility with current liquid-based cytology tests [72]. Ongoing research and clinical applications aim to enhance ovarian cancer prognosis using microbiome-based diagnostics.

**Table 1:** Overview of microbiome alterations in gut, peritoneum, cervicovaginal, ovarian cancer tissue, and serum samples from patients with ovarian cancer. Plus sign (+) and minus sign (−) indicate more and less abundant, respectively, in ovarian cancer patients or ovarian cancer cells compared to control.

Location	Microbiome	Relative Abundance
Gut	*Bacteroides* [75]	+
	*Prevotella* [75]	+
	*Proteobacteria* [75]	+
	*Ruminococcus* [75]	−
	Actinobacteria [75]	−
Peritoneum	18 microbial features [78]	Unique distribution
Ovarian Cancer Tissue	*Brucella* [38]	76% of patients
	*Chlamydia* [38]	60% of patients
	*Mycoplasma* [38]	74% of patients
	*Proteobacteria/firmicutes* [80]	+
	*Acinetobacter* [80]	+
	*Lactococcus* [80]	−
Cervicovagina	*Lactobacilli* [68]	−
	*Mobiluncus curtisii* [82]	+ → −
	*Eubacterium rectale* [82]	+ → −
	*Fusobacterium nucleatum* [82]	+ → −
	*Porphyromonas* [82]	+ → −
Serum	*Acinetobacter* [83]	+

### 3.2. Ovarian Cancer Treatment and Microbiome Effects

The treatment of ovarian cancer includes surgery and chemotherapy. These treatments were linked to the microbiome (Table 2). A previous study showed that the composition of the gut microbiome was altered after ovarian cancer surgery [84]. The relative proportion of *Proteobacteria* increased and the relative proportions of *Bacteroidetes* and *Firmicutes* decreased after surgery. These microorganisms are also associated with enteritis and colitis [85,86]. After surgery, the number of bacteria producing SCFAs, such as *Bacteroidetes*, *Faecalibacterium*, *Blautia*, *Roseburia*, and *Prevotella*, decreased. SCFAs have anti-inflammatory, anti-cancer, and immune effects [14,87]. Therefore, surgery can alter the microbiome composition, which can have various effects on the body.

Platinum-based anti-cancer drugs are commonly used to treat several cancers, including ovarian cancer. However, some studies have shown that these drugs affect the gut microbiome. One study found that certain bacteria, including *Bacteroides*, *Collinsella*, and *Blautia*, increased in number after several cycles of chemotherapy [84]. *Bacteroides* and *Collinsella* are associated with rectal cancer [88,89]. *Bifidobacterium* increased after one–three cycles of chemotherapy [84]. This bacterium plays a crucial role in maintaining the gut microbial balance and has been linked to anti-cancer effects [90].

Cyclophosphamide, in addition to platinum-based chemotherapy, is used to treat severe ovarian cancers. One study reported that it also affected the gut microbiome [91]. In this study, we found that mice treated with cyclophosphamide experienced a breakdown of the small intestinal epithelial barrier and a decrease in the number of *Lactobacilli* and *Enterococci* in the small intestine. This indicates that cyclophosphamide may facilitate the movement of bacteria across the intestinal epithelium and surrounding environment. The study found that bacteria present in the small intestine were also present in the mesenteric lymph nodes and spleen. This leads to the activation of the immune response, which increases helper and memory T cells, ultimately promoting anti-cancer effects. These results suggest that cyclophosphamide enhances anti-cancer effects by shifting the gut microbiome to lymphoid organs.

Studies have shown that the vaginal microbiome can affect chemotherapy in ovarian cancer [92]. Gemcitabine, a chemotherapeutic drug used to treat ovarian cancer, was less effective when tumor cells were cocultured with *Mycoplasma* [92]. This was attributed to the rapid degradation of gemcitabine by pyrimidine nucleoside phosphorylase and cytidine deaminase in *Mycoplasma*. Another study found that patients with platinum-resistant tumors were more likely to have a vaginal microbiome dominated by *Escherichia coli* [93].

Furthermore, the combination of surgery and chemotherapy affects the vaginal microbiome. A study on patients with ovarian cancer found that the presence of *Lactobacilli* decreased when both treatments were applied [75]. The authors attributed this to the dynamics among ovarian cancer, estrogen, and glycogen. Specifically, chemotherapy and an oophorectomy decrease estrogen production which, in turn, reduces glycogen in the vagina, leading to a decrease in *Lactobacilli*. As discussed in Section 2.2.1 and Section 2.2.3, a reduction in *Lactobacilli* may promote ovarian cancer progression.

Recent studies have revealed a link between the microbiome and ovarian cancer, leading to attempts to prevent or improve treatment efficiency by transplanting healthy microbes. There are two main types of microbiome transplantation: fecal microbiome transplantation (FMT) and vaginal microbiome transplantation (VMT).

Previous studies have explored the use of FMT for the treatment of other cancers [94,95,96,97]. However, to the best of our knowledge, only one study of FMT as a treatment has been directly relevant to ovarian cancer. Chambers et al. conducted a study which demonstrated that mice treated with antibiotics experienced accelerated ovarian cancer growth and increased cisplatin resistance [98]. However, when these mice were cecally transplanted with a microbiome derived from healthy mice, chemotherapy resistance was mitigated and their lifespan was prolonged. Although there are few studies, there is a glimpse into the potential for FMT in ovarian cancer, and further research could be beneficial for treatment.

No studies have directly addressed the link between VMT and ovarian cancer. Studies have shown that VMT reduces recurrence rates and improves symptoms in patients with bacterial vaginosis [99,100]. Therefore, VMT may be a plausible approach. Based on these findings, we propose investigating the use of VMT to modulate the cervicovaginal microbiome to increase drug responsiveness. This will improve the efficiency of ovarian cancer treatment.

**Table 2:** Overview of the relationship between the microbiome and the treatment of ovarian cancer.

Treatment Type	Microbiome	Contents
Surgical Therapy	*Proteobacteria* *Enterobacteriaceaea*	Increase in relative proportion after ovarian cancer surgery [84,101]
	*Bacteroidetes* *Firmicutes* *Faecalibacterium* *Blautia* *Roseburia* *Prevotella*	Decrease in relative proportion after ovarian cancer surgery [84]
Chemotherapy	*Bacteroides* *Collinsella* *Blautia*	Increase in relative proportion after platinum-based chemotherapy [84]
	*Lactobacilli* *Enterococci*	Relative proportion in small intestine decreased, and relative proportion in mesenteric lymph nodes and spleen increased after cyclophosphamide administration [91]
	*Bifidobacterium*	Increase in relative proportion after one to three cycles of chemotherapy [84]
	*Mycoplasma*	Mycoplasma’s enzymes rapidly break down gemcitabine, reducing its responsiveness to the drug [92]
Chemotherapy with surgery	*Lactobacilli*	Reduction in relative proportion after oophorectomy and chemotherapy [75]

## 4. Discussion and Conclusions

The pivotal role of the microbiome in maintaining human health is evident and dysbiosis has been associated with various diseases, including ovarian cancer. This review highlights the potential repercussions of changes in the gut microbiome, particularly intensifying the inflammatory responses and increasing the risk of endometriosis, thereby increasing the likelihood of ovarian cancer. The IL-6 and Hh signaling pathways are likely involved in the mechanism by which the gut microbiome increases ovarian cancer risk by intensifying the inflammatory response. However, a comprehensive understanding of how endometriosis contributes to ovarian cancer risk requires further research, particularly to elucidate the potential interaction between endometriosis and the gut microbiome, as evidence involving factors such as peritoneal macrophages and estrogen remains limited.

The association between Chlamydia and ovarian cancer risk is intricate, with potential mechanisms including DNA damage or the evasion of apoptosis. However, the possibility of a Chlamydia infection leading to PID and subsequently increasing the risk of ovarian cancer varies across ethnic groups and within the same group. Lifestyle, genetic factors, and research methodologies have been proposed as contributors to these divergent findings, emphasizing the need for further investigation to uncover the factors that influence how PID may lead to ovarian cancer. The relationship between *Gardnerella vaginalis*, *Lactobacillus iners*, and ovarian cancer risk, as well as the conflicting results regarding miRNA-223 expression in patients with EOC, necessitates additional exploration. Although studies on miRNAs in ovarian cancer have been conducted, the link between the microbiome and ovarian cancer remains underexplored.

The potential impact of *BRCA1/2* mutations on ovarian cancer risk by reducing *Lactobacilli* requires further research, particularly to reconcile the contradictory findings on the effects of progesterone. Establishing biomarkers for early ovarian cancer diagnosis is imperative for high survival rates, and the microbiome in the gut, peritoneum, ovarian cancer tissue, cervicovaginal area, and serum holds promise as diagnostic markers. However, research on the gut microbiome as a biomarker lags behind that on the cervicovaginal microbiome. Although *Mycoplasma* has been identified in ovarian cancer tumor cells, its impact on ovarian cancer risk remains inconclusive and requires further investigation. *Lactobacilli*, which exhibit a consistent decrease in the cervicovaginal microbiome of patients with ovarian, cervical, and precancerous diseases, may be a valuable biomarker, but should be used in combination with other biomarkers to enhance diagnostic accuracy.

In exploring the potential impact of the microbiome on ovarian cancer treatments, this review suggests a plausible influence of both surgical and chemotherapy treatments on the efficacy and adverse effects. Although the microbiome may play a role in the response to chemotherapy, the direct relationship between the microbiome and chemotherapy in ovarian cancer requires further investigation, especially considering the drugs commonly used to treat various cancers. The concept of preventing ovarian cancer through microbiome transplantation from healthy individuals into those at high risk, or improving treatment efficiency through microbiome transplantation in patients with ovarian cancer, underscores the potential connection between ovarian cancer and the microbiome. The limited direct applications of FMT and VMT in ovarian cancer emphasize the need for further exploration, despite encouraging outcomes in related studies. These findings suggest that investigating microbiome transplantation in ovarian cancer is promising and merits further research.
